# Safety of Excipients in Pediatric Formulations—A Call for Toxicity Studies in Juvenile Animals?

**DOI:** 10.3390/children2020191

**Published:** 2015-05-15

**Authors:** Georg Schmitt

**Affiliations:** F. Hoffmann-La Roche Ltd., Roche Pharmaceutical Research and Early Development, Roche Innovation Center Basel, Basel CH-4070, Switzerland; E-Mail: georg.schmitt@roche.com; Tel.: +41-61-688-7576; Fax: +41-61-688-8101

**Keywords:** pediatric drug development, pediatric formulations, safety, juvenile animal studies

## Abstract

The development of drug products for pediatric use often requires age-appropriate formulations which can be more complex and may involve a broader range of excipients than adult dosage forms. Excipients established for adult use are not always appropriate for use in children because they can affect children differently than adults. Therefore, a comprehensive safety assessment of the excipients in a pediatric formulation is essential before use, referring to existing safety data from adult human and animals as well as safety data from pediatric use and juvenile toxicity studies, when available. The overall risk assessment needs to consider the safety risk from the excipients and the extent to which the risk from the disease as such will be ameliorated by the drug formulation. Non-clinical safety studies in juvenile animals are used to assess for specific toxicities or sensitivities of excipients and for establishing safe exposures in pediatric age groups. As for any active ingredient, non-clinical safety studies in juvenile animals should only be performed for excipients if important for clinical risk assessment and labelling. Pharmaceutical companies should be critical of excessive demands for juvenile animal testing, particularly of excipients when critically needed for significant therapeutic benefit.

## 1. Introduction

The development of drug products for pediatric use often requires age-appropriate formulations. Excipients are essential components of drug formulations to overcome challenges such as solubility and stability. Masking of taste and smell is an important aspect particularly for oral pediatric formulations to ensure compliance in children and thus to guarantee therapeutic success. As a result, pediatric formulations may be more complex and may involve a broader range of excipients than adult dosage forms. For these reasons, the choice of suitable excipients is a key element in pediatric drug development [1]. There is no general approval process for excipients and they are approved together with a drug (as drug product) under particular settings (e.g., indication, route, dose-levels). Since excipients may also bear toxic potential there is a need to limit number and amount of excipients to the absolute minimum, *i.e.*, just as much as needed. Established excipients in drug formulations for adults may not be appropriate for use in children because they can affect children differently than adults. For example, the intake of an excipient may result in a higher exposure in children compared to adults, or to children of different ages, and may have different effects on developing organ systems, depending on age and developmental stage. Also the dosing accuracy of a drug product can be an issue for the safety of excipients in children. Therefore, each excipient selected for a pediatric formulation has to be justified in terms of safety for the targeted age group, taking into account knowledge about age specific characteristics and differences with respect to adverse effects, exposure, metabolism and elimination. In some cases, unique pediatric formulations may call for excipients with little or no human use data, thus calling for toxicology studies in animals, if necessary including juvenile animal models. As a result, the safety of excipients in pediatric formulations is nowadays a central topic for drug development and during interactions with Health Authorities.

## 2. When to Call Toxicity Studies in Juvenile Animals?

The non-clinical safety of drugs (*i.e.*, active ingredients) for pediatric use is widely regulated and guidelines on juvenile animal testing exist in Europe [2], the US [3] and Japan [4]. The basic guiding principles in the regional guidelines are comparable though not identical. Therefore, international efforts are underway to further clarify and harmonize when juvenile animal testing is considered informative and necessary for the safety of pediatric subjects, reflecting both active ingredients and excipients (Nonclinical Safety Testing in Support of Development of Pediatric Medicines, ICH guideline S11, under development).

Guidelines exist from various regulators which cover aspects of pediatric formulation development in general [5]. As for any active substance, the principles of risk–benefit also apply to excipients included in formulations. The selection of acceptable excipients for use in children is however subjected to even more stringent requirements than for use in adults. There is no reference list available on excipients generally considered safe for use in pediatric formulations. The regulator requests scientific rationale for the selection of excipients used in pediatric formulations and supportive safety data [6]. Additional non-clinical safety (*i.e.*, mainly toxicology) studies may be requested for excipients to address important clinical concern which cannot be assessed ethically or safely in children using existing animal and/or human data [7].

Safety studies in juvenile animals with exposures during critical periods of development are used to assess qualitative and quantitative differences between immature and adult stages. Such studies address potential differences in sensitivity to toxic effects and mechanism of action of excipients in the developing organism *vs.* in adults. The juvenile animal models are not only inflicted with the common difficulties of species to species translation but also with additional ambiguities to translate postnatal developmental stages across species. For example, the ontogeny of metabolizing enzymes and transporter proteins in postnatally developing animals and their corresponding development in humans is not always known, yet has a critical role in the design and interpretation of juvenile toxicology studies. In addition, technical and practical limitations need to be considered before embarking on juvenile animal models (e.g., can a juvenile animal be given the planned human dose/volume of an excipient).

Such non-clinical safety studies in juvenile animals can support establishing safe exposures in pediatric age groups. For example, the safety of Poloxamer 188 in pediatric patients, used as an excipient in the ready-to-administer subcutaneous drug dosage form was supported through an extensive series of non-clinical studies, including repeat-dose toxicity studies in adolescent/young adult rats and dogs. However, performing routine and standard juvenile toxicity studies as a box-ticking exercise should be prevented. Pharmaceutical companies should be critical of excessive demands for juvenile animal testing, particularly of excipients when critically needed for significant therapeutic benefit. The notions in pharmaceutical companies that a regulatory request always needs to be fulfilled or that the comparably small investment for an additional non-clinical study (of questionable or even no value) outweighs the effort to discuss with the authorities and the potential loss of time to market needs to be challenged for scientific and ethical (also animal welfare) reasons.

If a juvenile toxicity study is already performed to address concern of the active ingredient, the clinical pediatric formulation—whenever available—should be used to assess the safety of the excipients at the same time. Thus, the drug formulation is assessed in its entirety and there is no need for additional stand-alone animal testing of the excipients. Indeed, non-clinical data have shown that effects from an active ingredient which are relevant for pediatric safety can be exacerbated by an excipient, making such combined non-clinical testing likely more informative than stand-alone testing of active ingredient and excipients [8]. Excipients of specific concern may call for additional endpoints to be included in such juvenile animal toxicity studies. In exceptional cases additional stand-alone testing may be needed to better differentiate potential adverse effects caused by the excipient from those caused by the active ingredient.

Any novel excipient has to be characterized appropriately in the non-clinical setting before its use in a human drug product, including repeat-dose toxicity, genotoxicity and reproductive toxicity studies, and for carcinogenic potential when chronic administration is foreseen [9]. The International Pharmaceutical Excipients Council has proposed an evaluation procedure for excipients, including tiered toxicology testing, yet it does not refer to children [10]. Excipients which have been qualified non-clinically for adult use may require additional data from juvenile animal models to cover potential concern for specific age groups.

No matter if it concerns an active ingredient or excipient, a toxicity study in juvenile animals should only be performed if it is important for clinical risk assessment or labelling. Relevant sources of information must be exhausted before a call for non-clinical safety studies for excipients is justified:

- Guidelines for use of excipients (FDA, CHMP, ICH);
- Position and Opinion papers (e.g., CHMP);
- National databases for registered drug products (e.g., US: dailymed [11], UK: Electronic Medicines Compendium [12], Germany: Rote Liste [13] France: Vidal [14], Switzerland: Arzneimittelkompendium der Schweiz [15];
- Approved drug products (e.g., FDA inactive ingredients database [16]; one issue is that medicines authorized in Europe have to provide full details of the qualitative and quantitative composition in terms of excipients only since 2010 [17];
- Regulations on food, food additives and flavoring (e.g., European Food Safety Scientific Assessments (EFSA), Joint Expert committee on food additives (JECFA): US FDA GRAS list);
- Publicly available excipient databases (e.g., Safety and Toxicity of Excipients for Pediatrics (STEP) database [18,19].

Experts from formulation development, non-clinical and clinical safety should work together to make a comprehensive safety assessment of the excipients planned to be used in a pediatric formulation. Such safety assessment should consider total doses, (maximum) concentrations and volumes of the excipients, dosing regimen, duration of treatment, route of administration, as well as the indication and the (minimum) age groups.

It should be noted that previously authorized pediatric products may contain levels of excipients which are now no longer recommended today for use in children [20]. In other words, just because an excipient is approved in a pediatric drug formulation may not automatically qualify its safe use in another pediatric formulation, route or age group. In addition to the information sources listed above, toxicological information from indexed literature, relevant handbooks, and other databases (e.g., PharmaPendium, TOXNET) as well as internal data (non-published scientific evidence) should be used to conclude on the safe use of selected excipients in a planned pediatric formulation. When making reference to existing data on the safety of excipients it should also be kept in mind that excipients can have different grades, types and sources, which may also affect their safety.

Neonates are of particular concern as they are uniquely vulnerable to adverse reactions, and a wide range of excipients has been linked to be of potential harm in this young age group [21]. Knowledge of age-related differences in absorption, distribution, metabolism and elimination may allow the use of human physiologically-based pharmacokinetic (PBPK) models to guide on acceptable excipient levels. Such an approach is routinely used for predictions of active ingredient exposure in pediatric populations [22].

In the future, data sharing between the industries (pharmaceuticals, food, cosmetics) will hopefully identify a broader set of established excipients, qualified by extensive safety data in animals and/or by wide use in pediatric patients. Data sharing efforts are currently mainly limited to efforts from Lhasa Limited (Vitic Excipients project) and EuPFI (STEP database), and need to be expanded through increasing numbers of publications of existing tolerability data.

## 3. Conclusions

Excipients are necessary ingredients of drug products, particularly in pediatric formulations, and excluding an excipient will not always be appropriate. Established excipients in drug formulations for adults may not be appropriate for use in children because they can affect children differently than adults. Toxicity studies of excipients need to be performed in juvenile animals if important for clinical risk assessment and labelling. Even excipients which bear significant toxic potential for children may be acceptable after a rigorous risk assessment. Such an assessment needs to consider the safety risk from the excipients and the extent to which the risk from the disease as such will be ameliorated by the drug formulation. Pharmaceutical companies should question excessive demands for juvenile animal testing, even if excipients are critically needed for significant therapeutic benefit.
